# RBC-NOS-Dependent S-Nitrosylation of Cytoskeletal Proteins Improves RBC Deformability

**DOI:** 10.1371/journal.pone.0056759

**Published:** 2013-02-12

**Authors:** Marijke Grau, Sebastian Pauly, Jamal Ali, Katja Walpurgis, Mario Thevis, Wilhelm Bloch, Frank Suhr

**Affiliations:** 1 Department of Molecular and Cellular Sport Medicine, German Sport University Cologne, Cologne, Germany; 2 Centre for Preventive Doping Research/Institute of Biochemistry, German Sport University Cologne, Cologne, Germany; 3 The German Research Center of Elite Sport, German Sport University Cologne, Cologne, Germany; Goethe University, Germany

## Abstract

**Background:**

Red blood cells (RBC) possess a nitric oxide synthase (RBC-NOS) whose activation depends on the PI3-kinase/Akt kinase pathway. RBC-NOS-produced NO exhibits important biological functions like maintaining RBC deformability. Until now, the cellular target structure for NO, to exert its influence on RBC deformability, remains unknown. In the present study we analyzed the modification of RBC-NOS activity by pharmacological treatments, the resulting influence on RBC deformability and provide first evidence for possible target proteins of RBC-NOS-produced NO in the RBC cytoskeletal scaffold.

**Methods/Findings:**

Blood from fifteen male subjects was incubated with the NOS substrate L-arginine to directly stimulate enzyme activity. Direct inhibition of enzyme activity was induced by L-N5-(1-Iminoethyl)-ornithin (L-NIO). Indirect stimulation and inhibition of RBC-NOS were achieved by applying insulin and wortmannin, respectively, substances known to affect PI3-kinase/Akt kinase pathway. The NO donor sodium nitroprusside (SNP) and the NO scavenger 2-(4-carboxyphenyl)-4,4,5,5-tetramethylimidazoline-1-oxyl-3-oxide (cPTIO) were additionally applied as NO positive and negative controls, respectively. Immunohistochemical staining was used to determine phosphorylation and thus activation of RBC-NOS. As a marker for NO synthesis nitrite was measured in plasma and RBCs using chemiluminescence detection. S-nitrosylation of erythrocyte proteins was determined by biotin switch assay and modified proteins were identified using LC-MS. RBC deformability was determined by ektacytometry. The data reveal that activated RBC-NOS leads to increased NO production, S-nitrosylation of RBC proteins and RBC deformability, whereas RBC-NOS inhibition resulted in contrary effects.

**Conclusion/Significance:**

This study first-time provides strong evidence that RBC-NOS-produced NO modifies RBC deformability through direct S-nitrosylation of cytoskeleton proteins, most likely α- and β-spectrins. Our data, therefore, gain novel insights into biological functions of RBC-NOS by connecting impaired RBC deformability abilities to specific posttranslational modifications of RBC proteins. By identifying likely NO-target proteins in RBC, our results will stimulate new therapeutic approaches for patients with microvascular disorders.

## Introduction

Nitric oxide (NO) is an essential short-lived diffusible molecule that critically regulates central physiological mechanisms. Endothelium-derived NO has been shown to cause relaxation of smooth muscle cells under normoxic conditions through activation of soluble guanylyl cyclase (sGC) cascade leading to vasorelaxation [1]. Endothelium-derived NO can also directly act in the blood stream, where it influences the activity of platelets at the vessel surface [2].Besides its interaction with the heme moiety of certain proteins, NO can be oxidized to nitrite and nitrate. Nitrite has been shown to represent an endocrine bioavailable storage pool of NO that can be bioactivated under hypoxic conditions [3], [4], [5], [6], [7], [8]. This reaction called ‘hypoxic vasodilation’ ensures an increase of local blood flow and thus oxygen supply to metabolically active tissue. In vascular smooth muscle cells nitrite is converted to NO via the heme globin myoglobin [9]. It has also been suggested that NO generated by red blood cells (RBCs) may also contribute to hypoxic vasodilation, conceding a role for RBCs in local blood flow [10].

But NO also binds to reactive cysteine thiols [11], [12], [13]. This reaction, termed S-nitrosylation, represents an important post-translational protein modification [14], analogous to phosphorylation [15], and affects most classes of proteins. The formation of these so-called RSNOs has been held responsible for physiological processes regulating activity, turnover, subcellular localization, and molecular interaction of diverse proteins [16]. Dysregulation of S-nitrosylation is associated with a variety of pathophysiological conditions, including multiple sclerosis, pulmonary hypertension or Parkinsońs disease [11], [17]. In line with this, a lack of NO has been shown to be a hallmark of endothelial dysfunction [18], [19] contributing to atherosclerosis, arterial hypertension or diabetes [20] pointing to a central role of NO in human diseases. NO is enzymatically produced through NOS of which red blood cell NOS (RBC-NOS) has been found to represent an active and functional endothelial type NOS (eNOS) localized in the plasma membrane and the cytoplasm of RBCs [21], [22], [23]. RBC-NOS activity was shown to alter functional characteristics of RBCs, importantly increased deformability of RBCs [24]. RBC-NOS activation is promoted by phosphorylation of its serine1177 (Ser^1177^) residue [25], [24] while reduction of enzyme activity is associated with phosphorylation of serine116 and threonine495 residues [26]. We have recently shown that increased shear stress *in vivo* results in an activation of the phosphatidylinositol 3 (PI3)-kinase/Akt kinase pathway that in turn stimulates RBC-NOS by phosphorylation at Ser^1177^ [24]. The subsequently RBC-NOS-produced NO was essential to beneficially promote RBC deformability [24], [27]. Similar results have been obtained in *in vitro* studies showing that under defined shear stress conditions RBC-NOS phosphorylation at Ser^1177^ increases which resulted in increased NO production [28]. We also demonstrated that inhibition of PI3 kinase pathway reduced the phosphorylation level of RBC-NOSSer^1177^ and consequently NO production as well as RBC deformability [24]. Together, these data clearly demonstrate a causal relation between RBC-NOS activation and RBC deformability, but the underlying mechanisms through which RBC-NOS-synthesized NO regulates deformability still remain unclear.

To shed light on the potential mechanisms, we hypothesize in the present study that RBC-NOS-generated NO leads to S-nitrosylation of cytoskeletal proteins critically involved in the regulation of RBC deformability. Additionally, we establish RBC nitrite as a useful and easy accessible marker reflecting RBC-NOS activity.

## Methods

### Ethical approval

The protocols used in this study were approved by the ethics committee of the German Sports University Cologne. These protocols align with the Declaration of Helsinki and all participants gave written informed consent to participate in this study.

### Used reagents chemicals

The following reagents, chemicals as well as their applied concentrations/dilutions were used to perform the described experiments:

3,3-diaminobenzidine-tetrahydrochloride solution (Sigma, St. Louis, USA), acetic acid (Carl Roth, Karlsruhe, Germany), acetonitrile (Sigma-Aldrich, Steinheim, Germany), ammonium bicarbonate (100 mM; Sigma-Aldrich, Steinheim, Germany), ferricyanide (Sigma-Aldrich, Steinheim, Germany), hydrogen peroxide (Merck, Darmstadt, Germany), Igepal (Sigma-Aldrich, Steinheim, Germany), Insulin (200 pM; Gibco ® Life Technologies, Darmstadt, Germany), L-arginine (3 mM; Sigma-Aldrich, Steinheim, Germany), L-N5-(1-Iminoethyl)-ornithine (L-NIO; 10 µM; Axxora, Lörach, Germany), methanol (Carl Roth, Karlsruhe, Germany), milk powder (Bio Rad, Munich, Germany), N-ethylmaleimide (Calbiochem, Darmstadt, Germany), NO donor sodium nitroprusside (SNP; 100 µM; Alexis Biochemicals, Lörrach, Germany), NO scavenger 2-(4-Carboxyphenyl)-4,4,5,5-tetramethylimidazoline-1-oxyl-3-oxide (cPTIO; 300 µM; Cayman Chemical, Ann Arbor, USA), PageBlue™ Protein Staining Solution (Fermentas Life Science, St. Leon-Rot, Germany), paraformaldehyde (4%; Merck, Darmstadt, Germany), phosphate-buffered-saline (PBS; PAA, Pasching, Austria), Polyvinylpyrrolidone (PVP; 0.14 mM), primary antibody against eNOSSer^1177^ (dilution 1∶500; Upstate, Lake Placid, USA), secondary goat-anti-rabbit antibody (dilution 1∶400; Dako, Glostrup, Denmark), streptavidin-horseradish-peroxidase complex (dilution 1∶150; Amersham, Buckinghamshire, England), trifluoroacetic acid (Sigma-Aldrich, Steinheim, Germany), Tris-buffered saline (TBS, 0.05 mol, pH 7.6; consisting of Tris-hydroxymethylaminomethane, sodium chloride and hydrochloric acid, Merck, Darmstadt, Germany), trypsin solution (20 µg/ml in 50 mM NH_4_HCO_3_, pH 8; Promega, Mannheim, Germany), Wortmannin (10 µM; Sigma-Aldrich, Steinheim, Germany), XT Tricine running buffer (BioRad, Munich, Germany).

### Blood sampling

Venous blood was taken from the antecubital vein from 15 male subjects. Basal anthropometric parameters of the subjects were as follows (mean±SD): age [years]: 28.3±3.5, height [cm]: 181±6, weight [kg]: 78.6±10.8. All subjects were non-smokers, abstained from alcohol consumption for at least 24 hours prior to blood withdrawal and venous blood samples were taken under fasting conditions. Blood samples were anticoagulated using Heparin vacutainer (BD Vacutainer, Franklin Lakes, USA).

RBCs were separated from leukocytes and platelets by centrifugation (800 x g, 4°C, 10 min) and resuspended in autologous plasma to a hematrocrit of 40% for all experimental setups. All RBC preparations were carried out immediately after blood collection.

### Sample preparation

The RBC suspensions were divided into aliquots and incubated for 60 min at 37°C in the presence of PBS serving as control, L-arginine [22] as RBC-NOS substrate, and (L-NIO [29], [30], [31] as RBC-NOS inhibitor. Wortmannin has been shown to inhibit PI3 kinase, thereby decreasing Akt activity and consequently decreases phosphorylation of the eNOSSer^1177^ as well as RBC-NOSSer^1177^ [32], [24]. Wortmannin [24] was used to determine a direct link between PI3 kinase/Akt/RBC-NOS and NO signaling in human RBCs [24]. Insulin [22] has been shown to activate RBC-NOS by increasing its phosphorylation at serine^1177^ [22]. The NO donor SNP [33], [34] served as NO positive control and applied to analyze its effects on the examined parameters. The NO scavenger cPTIO [35] oxidizes NO to nitrite [36] and was therefore used as a negative control to study the effects of diminished NO content.

After 60 min incubation, RBCs were separated from plasma by centrifugation at 5,000 x g for 1 min and 4°C. Phosphorylation of RBC-NOSSer^1177^, RBC nitrite level, RBC deformability, and S-nitrosylation of RBC proteins were investigated in treated RBCs. Nitrite and N^G^,N^G^-dimethylarginine (ADMA) level were determined in plasma fractions. All measurements were carried out at 37°C with physiological pH 7.4.

### Measurement of endogenous NOS inhibitor ADMA

L-Arginine is found in the mammalian organism at concentrations by far exceeding K_M_ values of the NOS enzymes [37]. Therefore, additional L-arginine should not enhance NO formation. *In vivo*, however, increasing L-arginine concentration has been shown to increase NO production [38], [39]. This phenomenon has been termed as L-arginine paradox. ADMA has been shown to be an endogenous potent inhibitor of various NOS isoforms. Bound ADMA can be displaced by L-arginine which may serve as one explanation for increased NO production upon L-arginine supplementation [40], [41].

Plasmatic ADMA concentration was determined spectrophotometrically using the ADMA direct ELISA kit (Immundiagnostik, Bensheim, Germany). This assay is based on a peroxidase dependent oxidation step followed by a color change from blue to yellow. The absorbance was measured at 450 nm with the intensity of the yellow color being inverse proportional to the ADMA concentration. A dose response curve of absorbance unit vs. concentration was generated using the values obtained from the standard. The results were calculated using 4-parameter-algorithm.

### Immunohistochemical procedure

To determine the activation of the RBC-NOS after inhibition and stimulation of the PI3/Akt kinase signalling pathway, RBCs were fixed with 4% paraformaldehyde (v/v; 1/1) immediately after separation as described by Suhr et al. [26], [24]. Briefly, RBCs were dispersed on a slide and heat fixed. The slides were washed and RBCs were permeabilized in 0.1% trypsin, placed in a solution of 2% hydrogen peroxide and 80% methanol/TBS, and treated with 3% milk powder in 0.1 M TBS. The test area of each slide was incubated with a primary antibody against eNOSSer^1177^ while the control area was incubated in the absence of eNOSSer^1177^ antibody. The samples were incubated with a secondary goat-anti-rabbit antibody and as a detection system the streptavidin-horseradish-peroxidase complex was applied. The staining was developed using 3,3-diaminobenzidine-tetrahydrochloride solution in 0.1 M TBS.

Published protocols [22], [42] were used to analyze the intensity of immunostaining in RBCs [24]. The intensity of immunostaining is reported as the mean of measuring RBC gray value minus background gray value, which was detected at a cell-free area of the slide [24]. For staining intensity detection, a Leica microscope coupled to a CCD-camera (DXC-1850P, Sony, Germany) was used and the analysis was conducted using the software “Image J” (National Institutes of Health, Bethesda, Maryland, USA). Magnification for all images was 400-fold.

### Measurement of RBC and plasma nitrite

The nitrite level is well-known as an indicator of NO production under physiological and pathophysiological conditions [43], [44], [45] and is therefore of high relevance as a biochemical parameter. Therefore, we determined RBC and plasma nitrite in dependence of RBC-NOS activity, NO donors and inhibitors, respectively, according to the recently used protocols [46], [47]. After incubation, blood samples were separated by centrifugation as described above. Plasma samples were immediately snap-frozen and stored at −80°C until measurement. To preserve nitrite in RBCs, a ferricyanide-based preservation solution was added to the RBCs in a 1∶5 ratio (v/v; preservation solution/RBCs). The solution consists of 0.8 M ferricyanide, 0.1 M N-ethylmaleimide, and Igepal (10% of the total volume of preservation solution). The samples were mixed and snap-frozen in liquid nitrogen to yield a higher purification degree. For nitrite measurement in RBCs, methanol (VWR international, Darmstadt, Germany) was added to the frozen samples in a 1∶2 ratio to remove proteins. After centrifugation at 21,000 x g, 4°C and 15 min the nitrite level of the supernatant and of the thawed plasma samples were determined by injecting 100 µl into an acidified tri-iodide solution that reduces nitrite to NO gas. Along with helium gas-stream NO was purged into an ozone-based chemiluminescence NO detector (CLD 88 NO, Ecophysics, Switzerland) [48]. The Power Chrome software (Ecophysics, Switzerland) was used to analyze the area under the curve. All samples were measured in triplicate. Using aqueous calibration solutions with known nitrite concentration allowed calculation of sample nitrite content.

### S-nitrosylation assay, gel electrophoresis and western blot

NO was shown to bind to thiol (SH) groups of protein cysteine residues resulting in the formation of an S-NO moiety. This reaction, termed S-nitrosylation, is a reversible and post-translational modification that regulates the activity of a large number of targets, including structural, cytoskeletal and signaling proteins [49]. The used S-Nitrosylated Protein Detection Assay (Cayman Chamical, Ann Arbor, USA) was modified after the “Biotin-switch” method of Jaffrey et al. [50]. After incubation, separated RBCs were lysed and free SH groups were blocked. Any S-NO bonds present in the blood sample was then cleaved. Newly formed SH groups were biotinylated. Protein concentration of the samples was determined using the DC-Protein Assay Kit (BioRad, Munich, Germany). A total of 10 µg and 50µg protein, respectively, was loaded into the lanes of a 3–8% Tris-acetate gel (BioRad, Munich, Germany). Proteins were separated for 1h with constant 90 mA according to their charge and mass in a 1 x XT Tricine running buffer. One part of the gel containing 10µg of separated proteins was transferred to a polyvinylidene fluoride membrane (0.45 mm pore size) and the other part containing 50µg of separated protein per lane was stained using PageBlue™ Protein Staining Solution. The membrane was blocked in 2% bovine serum albumin (in 1x TBST), incubated with a horseradish peroxidase (dilution 1∶2000) and immunoreactive bands were developed by an enhanced chemiluminescence kit (Thermo Scientific, Darmstadt, Germany). Western blot bands were examined for differing intensities between the pharmacological treatments using software ‘Image J’ and the difference in intensity between intervention and control was calculated. Background of PageBlue™ stained gels was eliminated by distilled water. Bands that showed differing intensities in the western blot were excised from the gel to identify the corresponding protein by LC-MS/MS analysis.

### Bottom-up protein identification

The proteins of the altered bands were determined/analyzed by using bottom-up proteomic approaches including in-gel tryptic digestion and nano-liquid chromatography high resolution/high accuracy Orbitrap mass spectrometry as already described elsewhere [51]. In brief, the respective gel bands were excised from the PageBlue™-stained gel, destained by using 100 mM ammonium bicarbonate (NH_4_HCO_3_)/ acetonitrile (ACN; 1∶1) and dehydrated by incubation in ACN and under reduced pressure. After rehydration of the gel slices in a trypsin solution (20 µg/ml in 50 mM NH_4_HCO_3_, pH 8), proteins were digested over night at 37°C. Tryptic peptides were extracted using a mixture of 50% ACN/1% trifluoroacetic acid (TFA), dried in a vacuum centrifuge and finally reconstituted in 2% acetic acid. The analysis of the tryptic peptides was accomplished by using a LTQ Orbitrap high-resolution hybrid mass spectrometer (Thermo Fisher, Bremen, Germany) coupled to a Waters nano-Acquity UPLC system (Eschborn, Germany).The LC system was used in combination with a Symmetry C18 precolumn (particle size: 5 µm, 180 µm x 20 mm, flow rate 5 µL/min) and a BEH130C18 peptide column (particle size: 1.7 µm, 100 µm x 100 mm, flow rate 750 nL/min). The following gradient program with 0.1% formic acid as solvent A and ACN containing 0.1% formic acid as solvent B was used: 3 min 97% A (trapping operated at a flow rate of 5 µL/min), 3–5 min 80% A, 5–45 min 40% A, 45–48 min 20% A, 48–50 min 3% A, 50.01 min 97% A, 15 min equilibration with 97% A (analytical flow rate 750 nL/min). The LTQ Oribitrap MS was equipped with a nanospray ion source and operated in positive mode with an ionization voltage of 1.4 kV. Full scan spectra were recorded with a resolution of 30000 FWHM (at *m*/*z* 400) and data dependent MS/MS experiments fragmenting the three most abundant ions with a charge state > 1 were performed in the linear ion trap. For that purpose, helium was used as collision gas and the collision energy was set to 35% (arbitrary units, Xcalibur 2.1, Thermo Fisher). As a damping gas, nitrogen was obtained from a CMC nitrogen generator (CMC Instruments, Eschborn). The resulting MS data were evaluated using Proteome Discoverer Software (Thermo Fisher, Version 1.0, 2008), SEQUEST algorithm and the human_ref.fasta database (2006). The identification of a protein was considered successful if at least two peptides fulfilling a high level of confidence (strict FDR: 0.01) were detected or the sequence coverage was above 10%.

### RBC deformability

RBC deformability was determined after incubation at various fluid shear stresses by laser diffraction analysis using the laser assisted optical rotational red cell analyzer (LORRCA, RR Mechatronics, Netherlands) according to the protocol of Suhr et al. [24]. The system has been described in detail elsewhere [52]. RBCs were mixed with an isotonic viscous medium (0.14 mM Polyvinylpyrrolidone (PVP), osmolality 300 mOsmol*L-1, viscosity 30 mPa*s at 37°C; Mechatronics, Netherlands) in a 1∶250 ratio. The blood/PVP sample was sheared in a Couette system. A laser beam was directed through the sheared sample and the diffraction pattern produced by the deformed red cells was analyzed by a computer. On the basis of the geometry of the ellipitical diffraction pattern, an elongation index (EI) was calculated: EI = (L−W)/(L+W), where L and W represent the length and width of the diffraction pattern, respectively. EI values were calculated for nine shear rates (0.3, 0.57, 1.08, 2.04, 3.87, 7.34, 13.92, 26.38, and 50 Pa) and all samples were measured in duplicate. Mean EI values were plotted as a function and according to Baskurt et al. [53], the Lineweaver-Burke equation was used to calculate the maximum deformability (EImax).

### Statistical Analysis

Statistical analyses of the data were performed by using statistics software packages (Origin 8.5 Pro, Northampton, USA and GraphPadPrism 4, La Jolla, USA). Data were analyzed by multi-way analysis of variances (ANOVA). Descriptive statistics of the data is presented as mean±standard error of means (S.E.M.) unless described otherwise. Statistical differences were considered to be significant for values of *P*<0.05.

## Results

### Activation of RBC-NOSSer^1177^

Studies have shown that phosphorylation of the serine1177 residue increases RBC-NOS enzyme activity [25], [28], [24]. Determination of RBC-NOSSer^1177^ therefore represents a reliable readout to examine RBC-NOS activation after incubation with various pharmacological agents.

**Figure 1:**
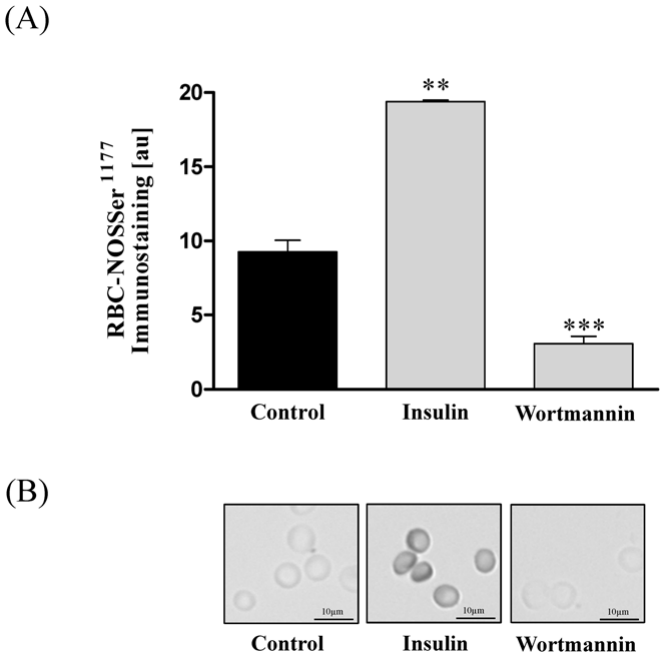
Changes of RBC-NOSSer^1177^ levels after RBC incubation with RBC-NOS stimulants, inhibitors and NO controls. (A) Bars show statistical analysis of gray values [au] after a 60 min incubation of RBCs with insulin and wortmannin, respectively. Application of RBC-NOS stimulant insulin led to a significant increase in enzyme phosphorylation compared to control (*P*<0.01). Wortmannin (*P*<0.001) application decreased phosphorylation, respectively. (B) Pictures show representative RBC-NOSSer^1177^ staining after 60 min incubation, respectively. Magnification for all images was 400-fold. Data in (A) are presented as mean±S.E.M., n = 6.

The statistical analysis of gray values against phosphorylated RBC-NOSSer^1177^ revealed that insulin increased phosphorylation of RBC-NOS at serine1177 from 9.5±0.41 au in control samples to 19.06±0.38 (*P*<0.01). Staining intensity of RBC-NOSSer^1177^ was significantly decreased after wortmannin (3.1±0.46; *P*<0.001) application. (Figure 1 A+B).

### Nitrite measurements as sensitive marker for RBC-NOS dependent NO synthesis

**Figure 2:**
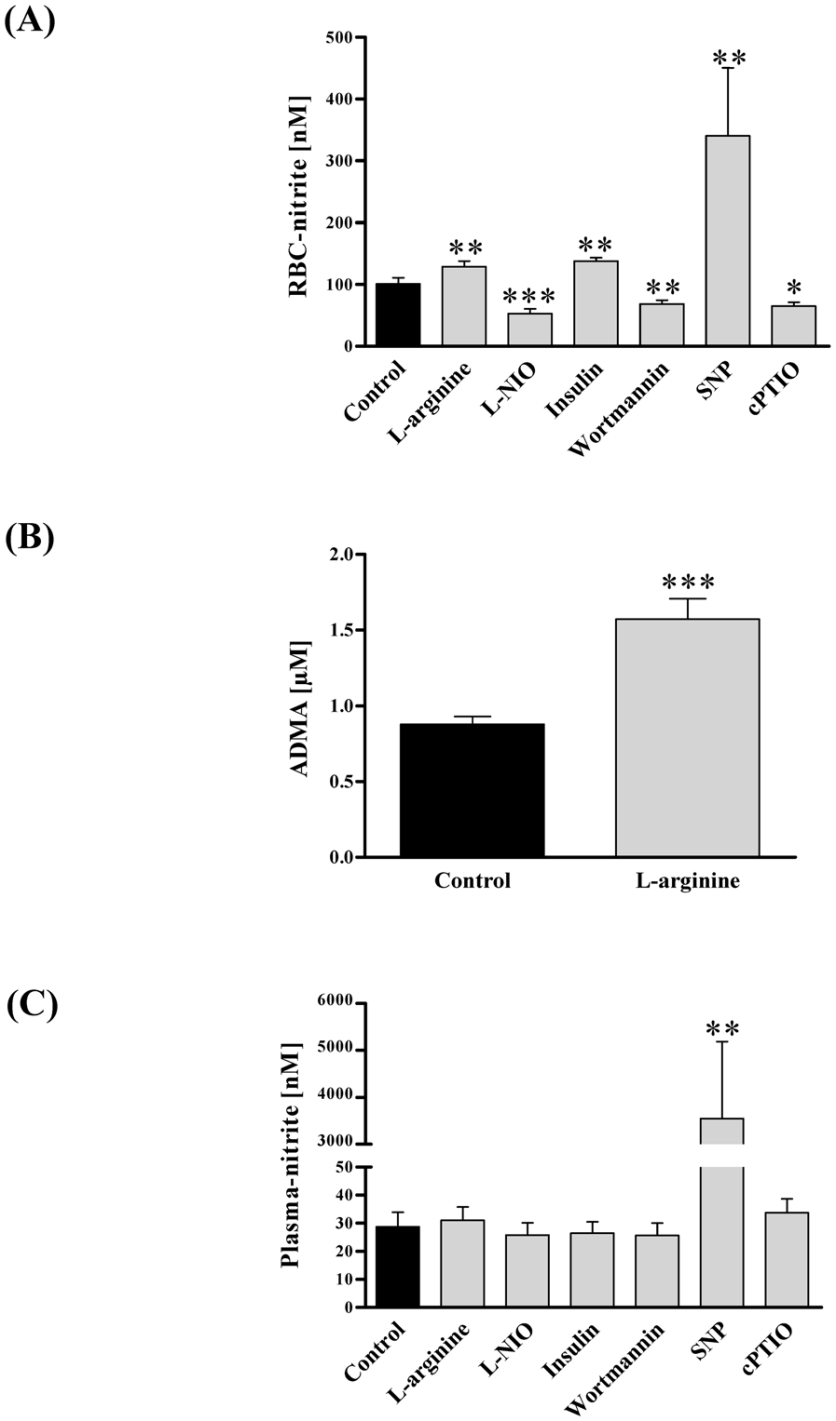
Changes in nitrite concentration after modification of RBC-NOS activity, and plasma ADMA content after L-arginine application. Nitrite was measured as representative of NO synthesis after 60 min incubation of RBCs with RBC-NOS stimulants, inhibitors and NO controls. (A) RBC nitrite level significantly increased after L-arginine (*P*<0.01), insulin (*P*<0.01) and SNP (*P*<0.01) application, while L-NIO (*P*<0.001), wortmannin (*P*<0.01) and cPTIO (*P*<0.05) significantly decreased RBC nitrite content compared to control samples. (B) Plasma ADMA concentration was additionally measured after L-arginine incubation to present an explanation for the L-arginine paradox. Bars show that ADMA concentration in the plasma fraction significantly increased upon substrate addition (*P*<0.001). (C) Plasma nitrite concentration was not altered by the applied substances, except SNP. Data in (A–C) are presented as mean±S.E.M., n = 15.

Changes of plasmatic nitrite correlates with acute alterations of eNOS activity in humans [43], [44], [45]. As RBC-NOS activity is not exclusively indicated by phosphorylation of the enzyme we determined RBC-NOS activity by additional measurements of plasma and RBC nitrite content thereby establishing RBC nitrite as a marker reflecting RBC-NOS activity. RBCs were incubated in the presence of both RBC-NOS stimulants and inhibitors. RBC nitrite increased from 100.9±10 nM in control samples to 129.1±9 nM (*P*<0.01) after L-arginine and to 138.0±5.5 nM (P<0.01) after insulin application, respectively, and decreased to 53.3±7.4 nM (*P*<0.001) after L-NIO and to 68.5±6.5nM (*P*<0.01) after wortmannin treatment, respectively. Addition of NO donor SNP increased RBC nitrite to 340.8±109.8nM (*P*<0.01) and NO scavenger cPTIO decreased nitrite to 65.1±6.5 nM (*P*<0.05; Figure 2A).

The increase of NOS enzyme activity through increasing the substrate concentration above the Michaelis constant is referred to as L-arginine paradox. Therefore, the concentrations of NOS inhibitor ADMA was measured in the plasma fraction to elucidate whether the significant increase in RBC nitrite after L-arginine incubation may be explainable by decreasing intracellular ADMA-enzyme binding as suggested by Tsikas and colleagues [54]. Our data revealed that the plasmatic ADMA content increased from 0.88±0.05 µM to 1.57±0.14 µM (*P*<0.001) after L-arginine incubation (Figure 2B).

Plasma nitrite level was not affected by either RBC-NOS stimulating or inhibiting agents. In control samples plasma nitrite level was 28.7±5.2 nM. After application of L-arginine and insulin plasma nitrite levels were 31.0±4.8 nM and 26.5±4.0 nM, respectively. L-NIO- and wortmannin-treated samples showed nitrite levels of 25.8±4.3 nM and 25.7±9.7 nM, respectively. In SNP and cPTIO treated samples nitrite levels of 3548.4±1637.5 nM (*P*<0.01) and 33.7±4.9 nM were measured (Figure 2 C).

### S-nitrosylation of red blood cell proteins after modulation of RBC-NOS activity

S-nitrosylation of RBC proteins was investigated after pharmacological stimulation and inhibition of RBC-NOS. Protein S-nitrosylation is a major mechanism determining the ubiquitous cellular properties of NO. Disruption of S-nitrosylation is associated with a wide range of pathophysiologic conditions [55], indicating the importance of cellular NO for tissue homeostasis.

**Figure 3:**
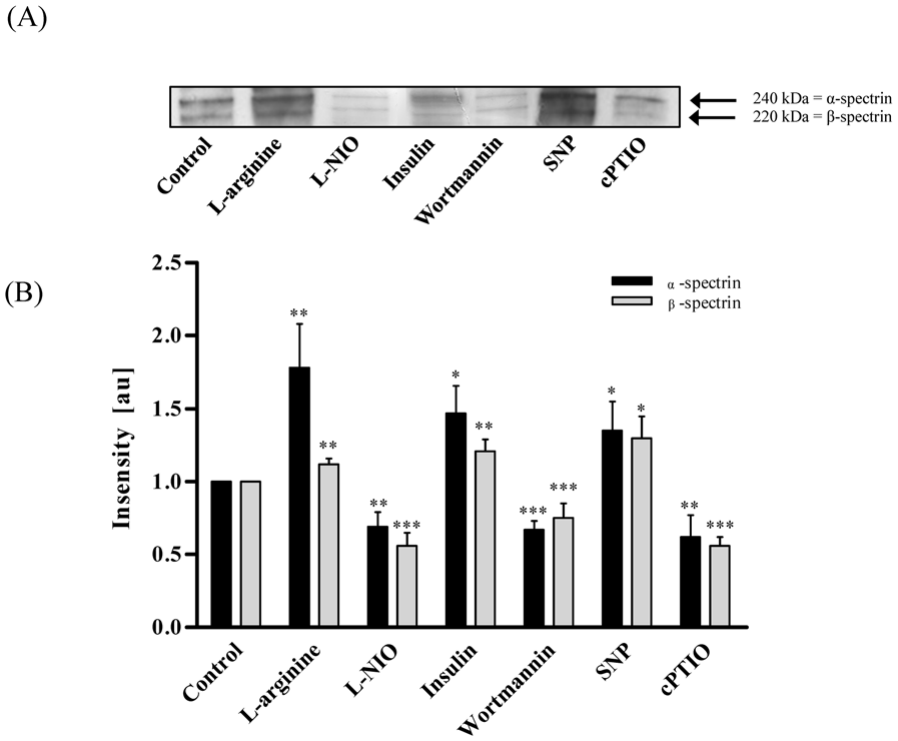
S-nitrosylation of RBC proteins after modification of RBC-NOS-dependent NO production. (A) Representative western blot bands after 60 min incubation of RBCs with RBC-NOS stimulants, inhibitors and NO controls are demonstrated. The image shows two distinctive bands with varying intensity depending on RBC-NOS modulation. LC-MS/MS analysis identified 240 kDa band as possible α- spectrin and 220 kDa band as possible β-spectrin. (B) Calculation of relative intensity in relation to the control sample revealed that S-nitrosylation of both, α- and β-spectrin, significantly increased upon L-arginine, insulin and SNP incubation and significantly decreased upon L-NIO, wortmannin and cPTIO incubation, respectively. Data in (B) are presented as mean±S.E.M., n = 6.

By western blot (WB) analysis we observed two distinct bands that were altered by RBC-NOS inhibition and stimulation as well as by addition of NO scavenger and NO donor. Using LC-MS/MS analysis these bands were identified. The first four proteins with the highest score are shown in table 1. Out of these, α-spectrin (240 kDa) and β-spectrin (220 kDa) showed the highest number of peptides identified and also the highest score; a value representing how well the measured mass spectrometry data align with the simulated protein data. Calculation of WB band intensity in relation to the control sample revealed that L-arginine, insulin, and SNP significantly increased S-nitrosylation of the protein, which most like represents α-spectrin, to 1.78±0.3 au (*P*<0.01), 1.47±0.19 au (*P*<0.05) and 1.35±0.2 au (*P*<0.05), respectively. Inhibition of RBC-NOS with L-NIO and wortmannin and application of NO scavenger cPTIO decreased S-nitrosylation of this protein to 0.69±0.1 au (*P*<0.01), 0.67±0.06 au (*P*<0.001) and 0.62±0.15 (*P*<0.01), respectively. Calculation of WB band intensity in relation to the control sample revealed that S-nitrosylation of the protein, which most like represents β-spectrin, showed highly comparable results (Figure 3A). Upon addition of L-arginine, insulin and SNP, intensity increased to 1.12±0.04 (*P*<0.01), 1.21±0.08 (*P*<0.01) and 1.3±0.15 (*P*<0.05), respectively. After addition of L-NIO, wortmannin and cPTIO, intensity significantly decreased to 0.56±0.09 (*P*<0.001), 0.72±0.05 (*P*<0.001) and 0.56±0.06 (*P*<0.001), respectively (Figure 3 B).

**Table 1:** MS data evaluation using Proteome Discoverer 1.0.

Western Blot bands	Accession number	Coverage (%)	# peptides	Score	Description
240 kDa	gi4507189	14.90	33	227.50	α-spectrin
	gi67782319	10.15	18	132.19	β-spectrin
	gi4507021	7.79	5	32.94	Solute carrier family 4
	gi4504345	21.83	2	14.88	α 2 globin
220 kDa	gi67782319	19.56	36	287.61	β-spectrin
	gi70780353	8.84	12	71.47	Ankyrin 1, isoform 4
	gi4507021	6.26	4	39.78	Solute carrier family 4
	gi4507189	1.48	3	13.94	α-spectrin

Excised and processed proteins were identified. The first four matches with their respective value concerning sequence coverage, number of peptides identified and score are presented.

### Maximum RBC deformability depends on RBC-NOS activity

**Figure 4:**
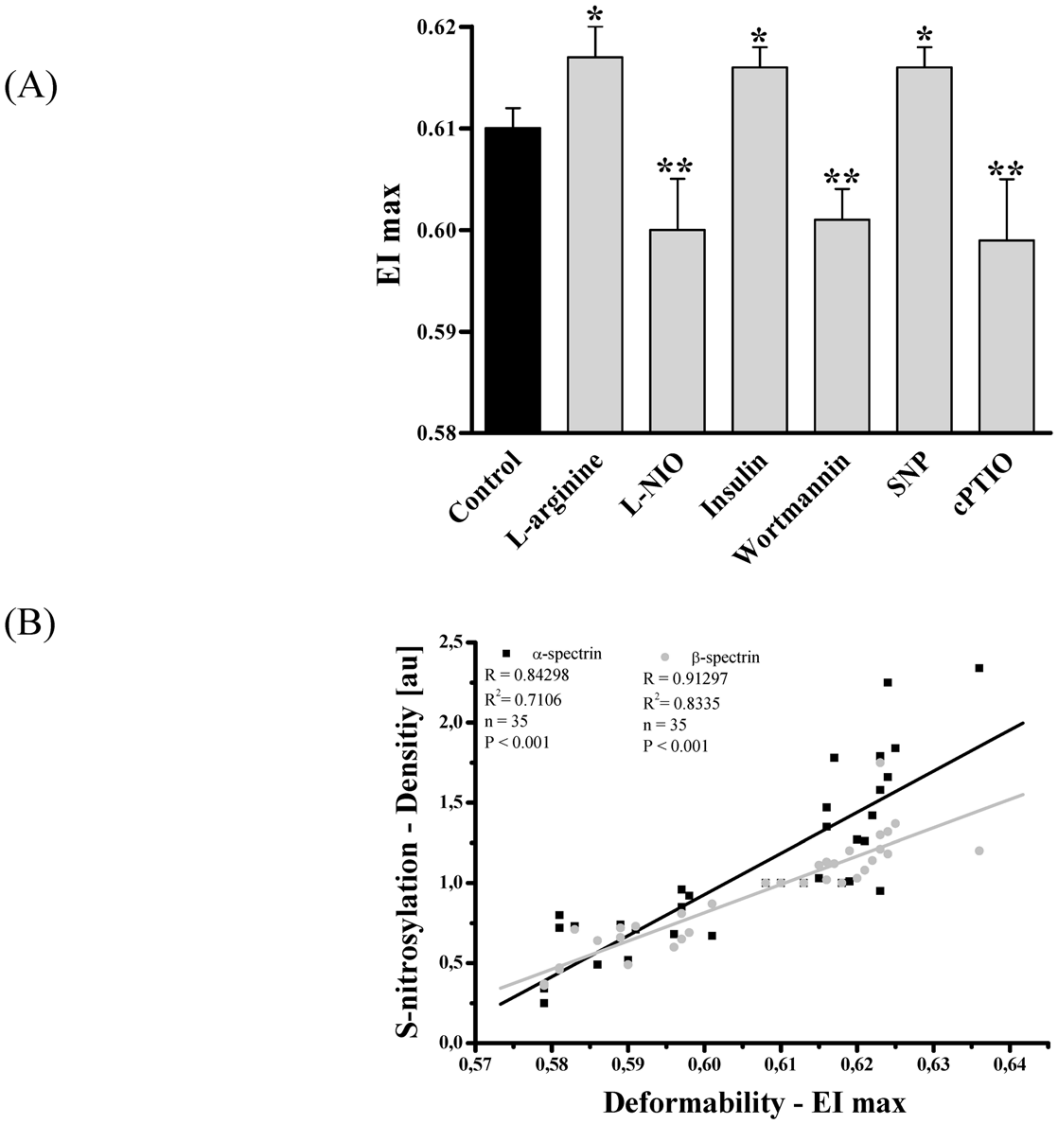
Maximum deformability (EI max) after RBC incubation with RBC-NOS stimulants, inhibitors and NO controls and correlation of EI max with S-nitrosylation of α- and β-spectrin. (A) Bars show that EI max significantly increased after 60 min incubation with L-arginine (P<0.05), insulin (P<0.05) and SNP (P<0.05). Application of L-NIO (P<0.01), wortmannin (P<0.01) and cPTIO (P<0.01) significantly decreased EI max. (B) Correlation between EI max and S-nitrosylation of α-(black square) and β-(gray circle) spectrin. Data in (A) are presented as mean±S.E.M of n = 15.

RBC deformability is crucial for the passage of RBCs through the capillary bed [56], [57]. Maximum deformability (EI max) was determined after pharmacological alteration of RBC-NOS activity. Our data showed that application of NOS stimulants L-arginine and insulin increased EI max from 0.61±0.002 in control samples to 0.617±0.003 (*P*<0.05) and 0.616±0.002 (*P*<0.05), respectively. The NOS inhibitors L-NIO and wortmannin significantly decreased EI max to 0.6±0.005 (*P*<0.01) and 0.601±0.003 (*P*<0.01), respectively. Addition of NO donor SNP increased EI max to 0.616±0.002 (*P*<0.05) and NO scavenger cPTIO decreased EI max to 0.599±0.006 (*P*<0.01; Figure 4 A). To determine a direct link between S-nitrosylation and deformability we have calculated a correlation from all random samples and reagents. Correlation of likely α-spectrin/values obtained from the 240 kDa band with EI max was calculated to be R = of 0.84 and R^2^ = 0.7106 with a significance of *P*<0.001. Correlation of likely β-spectrin/values obtained from the 220 kDa band with EI max revealed an R value of 0.91 and R^2^ = 0.8335 (*P*<0.001; Figure 4B).

## Discussion

It is generally accepted that the regulation of the peripheral vascular bed depends on endothelial NOS-mediated NO generation and –mediated signaling [58]. NO production by RBCs has been attributed to a NOS protein called RBC-NOS. The biological activity of this enzyme has been doubted in several studies [59], [60], but Kleinbongard et al. [22] and recently Cortese-Krott et al. [23] demonstrated that the RBC-NOS represents a functionally NOS protein that actively produces NO under normoxic conditions. Our group has also demonstrated that the activated RBC-NOS enzyme is critically responsible for NO production in RBCs and, thus, contributes to improved physical characteristics of RBCs, such as increased deformability [24].

These observations are of high relevance, as they let assume that patients suffering from diseases that reduce RBC deformability can be treated with specific RBC-NOS-activating pharmaceuticals to improve RBC deformability and consequently reduce the disease symptoms. However, in order to understand the precise effects of RBC-NOS-mediated improvements of RBC deformability, it is of great interest to be aware of the underlying molecular mechanisms resulting in improvements of RBC deformability.

This puts the cytoskeleton and the membrane of RBCs into focus of interest, which are composed of variety of molecules, including lipids, proteins, and carbohydrates [61]. These entire molecules act in concert to maintain the integrity of RBCs. Membrane and cytoskeletal proteins are strongly developed in RBCs, because they are mainly responsible for the resistance of RBCs against mechanical deformations as they occur during the passage of RBCs through small capillaries. It was demonstrated recently that RBC membrane lipids, such as cholesterol or adducin, have important regulatory functions in RBC deformability [62], [63]. Thereby, cholesterol increases fluidity and, thus, improves the viscosity of RBCs in patients suffering from hypertension [64]. However, it is well documented that RBC deformability is strongly reduced in patients with hypercholesterolemia [62]. In addition, it was postulated by Tziakas and colleagues [65] that increased content of RBC membrane cholesterol correlates with clinical instability of acute coronary syndrome patients. Adducin is responsible for the bridging of spectrin-actin junctions to band 3, which is the major RBC membrane-spanning protein in the lipid bilayer [63]. Therefore, adducin has an important role in RBC deformability, as the rupture of this bridge results in RBC membrane instability. Taken together, these data underline the importance of RBC membrane proteins in RBC deformability and impairments of these proteins result in clinical pathologies. However, the role of RBC membrane cytoskeletal proteins remains unknown to a large extent. Peripheral membrane proteins of RBCs located on the cytoplasmic surface of the lipid bilayer constitute the membrane skeleton, why especially these proteins determine the RBCs’ deformability and stability. A variety of peripheral membrane proteins have been identified in RBCs, of which the α- and β-spectrins play central roles [61], as a basic unit of the membrane skeleton consists of hexagonal lattices with six spectrin molecules.

Because the data from the literature point to an important role of cytoskeletal proteins in RBC deformability, we hypothesized that RBC-NOS-generated NO results in increased RBC deformability by directly mediating cytoskeletal proteins, most likely α- and β-spectrins. To address this hypothesis, we used *ex vivo* experimental setups. RBCs were isolated from blood of healthy male donors and incubated with a variety of different NOS activators and inhibitors. To specifically unravel the precise effect of RBC-NOS-generated NO in cytoskeletal modifications and, thus, in deformability, we used a variety of established and validated direct and indirect RBC-NOS inhibitors and stimulators, respectively. In detail, direct stimulation and inhibition were induced by RBC-NOS substrate L-arginine and substrate antagonist L-NIO. Indirect stimulation and inhibition were performed by adding insulin and wortmannin, respectively. Thereby, both reagents affect PI3 kinase/Akt kinase pathway which has been shown to influence RBC-NOS phosphorylation at Ser^1177^ [22], [24]. Additionally, we applied an NO donor and scavenger, respectively, to verify that the observed changes were caused by NO. By detecting RBC-NOSSer^1177^ immunohistochemically we clearly show that RBC-NOS in isolated RBCs can specifically be activated or inhibited. These data correspond to findings from *in vivo* experiments [24], indicating that our *ex vivo* approach reflects physiological RBC-NOS behavior. To measure the consequence of RBC-NOS activation we determined RBC-nitrite levels. Because NO is a highly diffusible and short-lived molecule [66], it is hardly possible to measure it directly. But NO can reliably be measured as nitrite, which is formed by oxidation of NO with a 1∶1 stoichiometry and, thus, serves as an important indirect marker for NO production [66]. We observed increased RBC-nitrite levels in RBCs that were treated with RBC-NOS activators but no changes in plasma nitrite. These data show that the activation of RBC-NOS resulted in a functionally increased circulating NO pool in RBCs. We also observed that RBC-NOS, like eNOS [41], may be inhibited by ADMA which is associated with impaired endothelium-dependent vasodilation. The present results show that L-arginine supplementation leads to increases in plasma ADMA concentration thereby reducing inhibition of the RBC-NOS and increasing NO generation and RBC deformability.

NO is not only oxidized to nitrite but also binds to free thiol groups of RBC proteins forming S-nitrosothiols. The influence of increased NO production on S-nitrosylation pattern of RBC proteins was detected by a specific S-nitrosylation assay [50] followed by gel electrophoresis, and identification of affected proteins was conducted by LC-MS/MS analysis. By means of these techniques we identified α- and β-spectrins as most likely targets of S-nitrosylation in RBCs. Furthermore and importantly, we demonstrate that the S-nitrosylation of these targets is directly caused by RBC-NOS activity and NO production. Incubation of RBC-NOS inhibitors resulted in decreased S-nitrosylation patterns of α- and β-spectrins, which were increased by RBC-NOS activators. It is of high relevance to underline that insulin incubation of RBCs resulted in increased S-nitrosylation patterns of both α- and β-spectrins. In terms of pharmacological treatment of diabetic patients and because of the finding that diabetic patients suffer inter alia from reduced RBC deformability, our data point to a molecular mechanism by that diabetic patients could benefit from insulin substitutions.

These data clearly show that RBC-NOS-generated NO plays an important role maintaining RBCs function, namely the post-translational modification of RBC membrane proteins. However, these data do not show whether the observed modifications exert functional and physiological consequences. We addressed this question by determining the RBC deformability after treatment of RBCs with RBC-NOS activators and inhibitors that resulted in increased and decreased RBC deformability, respectively. These data are in line with our previous *in vitro* experiments demonstrating that RBC-NOS-produced NO is responsible for increased RBC deformability [24]. Additionally, by correlation analysis we observed that the improvement of RBC deformability strongly depends on the S-nitrosylation status of α- and β-spectrins.

Taken together, our *ex vivo* data clearly demonstrate by unraveling the molecular mechanism that NO produced in RBCs has an important physiological function, as it mediates S-nitrosylations of central RBC membrane proteins. These data are the first to show that RBC-NOS-derived NO directly modifies RBC cytoskeletal proteins resulting in beneficial effects on RBC deformability. Therefore, this novel molecular mechanism responsible for RBC deformability opens up new strategies for the improvement of cardiovascular treatments, as a variety of cardiovascular diseases go along with impaired RBC deformability.
